# Mitochondrial Metabolism, Contact Sites and Cellular Calcium Signaling: Implications for Tumorigenesis

**DOI:** 10.3390/cancers12092574

**Published:** 2020-09-10

**Authors:** Roberta Peruzzo, Roberto Costa, Magdalena Bachmann, Luigi Leanza, Ildikò Szabò

**Affiliations:** Department of Biology, University of Padova, 35131 Padova, Italy; roberta.peruzzo@unipd.it (R.P.); robertocosta@live.it (R.C.); magdalena.bachmann@studenti.unipd.it (M.B.); ildi@bio.unipd.it (I.S.)

**Keywords:** mitochondria, contact sites, signaling

## Abstract

**Simple Summary:**

Several important cellular functions are finely tuned by the physical and functional interactions between mitochondria and other organelles, such as lipid trafficking, mitochondrial dynamics, calcium flow and Endoplasmic Reticulum stress. These functions in turn impact on apoptosis, autophagy, cell proliferation and differentiation, playing an important role in the pathogenesis of various human diseases. Mitochondria are closely interconnected with several organelles, such as Endoplasmic Reticulum, lipid droplets, Golgi apparatus, lysosomes, melanosomes and peroxisomes, through physical contacts. Several findings demonstrated that these interaction sites are important to fulfill specific cellular functions. In this review, we will highlight the role of membrane contact sites with mitochondria in modulating mitochondrial metabolism and intracellular signaling in the context of cancer development and progression, with a special focus on calcium signaling. In particular, we will discuss on mitochondria–ER, mitochondria–lysosomes and mitochondria–peroxisomes contact sites.

**Abstract:**

Mitochondria are organelles that are mainly involved in the generation of ATP by cellular respiration. In addition, they modulate several intracellular functions, ranging from cell proliferation and differentiation to cell death. Importantly, mitochondria are social and can interact with other organelles, such as the Endoplasmic Reticulum, lysosomes and peroxisomes. This symbiotic relationship gives advantages to both partners in regulating some of their functions related to several aspects of cell survival, metabolism, sensitivity to cell death and metastasis, which can all finally contribute to tumorigenesis. Moreover, growing evidence indicates that modulation of the length and/or numbers of these contacts, as well as of the distance between the two engaged organelles, impacts both on their function as well as on cellular signaling. In this review, we discuss recent advances in the field of contacts and communication between mitochondria and other intracellular organelles, focusing on how the tuning of mitochondrial function might impact on both the interaction with other organelles as well as on intracellular signaling in cancer development and progression, with a special focus on calcium signaling.

## 1. Introduction

Primordial mitochondria were symbiotic resident proteobacteria of the cell, providing an efficient energetic supply due to their ability to generate ATP using molecular oxygen as a final electron acceptor during respiration. As their counterpart, the host gives these ancient bacteria protection from the external environment and constant support in term of nutrients (e.g., ADP, amino acids, lipids and sugars) [1]. Primordial interactions with the host substantially differed from the current ones, because metabolic adaptation has constantly occurred within the cells during evolution. Indeed, mitochondria have lost about 90% of the original archaea-like genome, and most functions were transferred to the host nucleus [2]. Thus, cells acquired the ability to modulate mitochondrial metabolism; vice versa, mitochondria constantly communicate to the nucleus their energetic state via retrograde signals. Recently, the complexity of this communication has been further enriched in terms of organelles’ interaction. Mitochondria are connected to some intracellular compartments, and via these interactions they indirectly modulate some “a priori” independent biologic events (e.g., Wnt signaling or apoptosis) [3,4]. These facts drastically improve mitochondria-related functions, independently of the ancestral genome of mitochondria. Indeed, mitochondria are involved not only in cellular respiration but ATP and ROS production, both of which are generated during oxidative phosphorylation and also regulate cell differentiation, apoptosis, cell growth and cell cycle [5]. The participation of mitochondria in these cellular processes seems to depend also on retrograde signals, which allow the triggering of a transcriptional program that impacts on both organelle function and intracellular signaling [6].

Several important cellular functions are finely tuned by the physical and functional interactions between mitochondria and other organelles, such as lipid trafficking, mitochondrial dynamics, calcium (Ca^2+^) flow and ER stress. These functions in turn impact on apoptosis, autophagy [7] and melanogenesis [8]. The relevance of these inter-organellar contacts is underlined by the findings that mitochondria associated membranes (MAMs) seem to play an important role in the contexts of various pathologies, such as neurodegenerative diseases [9,10,11], diabetes [12], metabolic syndrome [13] and cancer [14].

Mitochondria are closely interconnected with several organelles by establishing physical contacts; for example, mitochondria have been proven to physically interact with Endoplasmic Reticulum (ER), lipid droplets, Golgi apparatus, lysosomes, melanosomes and peroxisomes [8,15]. Recently, system-level spectral imaging has revealed that even more than two organelles can interact at the same time, similar to what happens in plant cells performing photorespiration, where a physical contact between chloroplasts, mitochondria and peroxisomes allows metabolic coupling among all three organelles [16]. In particular, in the mammalian cells, mitochondria predominantly interact with ER, but then they contact Golgi, peroxisomes and lipid droplets [17]. These observations support the idea that these sites of physical interactions are important for specific cellular functions shared by different organelles, like mitochondria, ER and Golgi, all involved in cholesterol synthesis and transport [18].

In this review, we will discuss the role of mitochondria–organelle contact sites in modulating mitochondrial metabolism and intracellular signaling in the context of cancer development and progression. In particular, we will focus on mitochondria–ER, mitochondria–lysosomes and mitochondria–peroxisomes contact sites.

## 2. Mitochondria and Endoplasmic Reticulum

**Figure 1:**
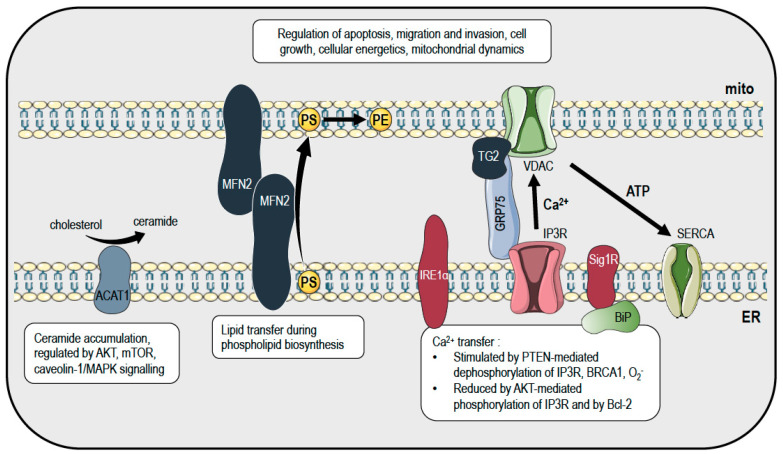
Cancer-relevant interactions between mitochondria and the ER at mito–ER contact sites (MERCs). Calcium transfer from the ER to mitochondria through the IP3R–GRP75–VDAC complex modulates mitochondrial energy metabolism and apoptosis induction, and is regulated by post-translational modifications of IP3R, ROS and associations with proteins such as TG2 and BRCA1. Further, proteins such as IRE1α and the Sig1R-BiP complex can interfere with the calcium transfer. Mitochondrial ATP, on the other hand, is important for the correct functioning of SERCA, and thus ER calcium homeostasis. These mechanisms influence, amongst others, cancer cell proliferation, invasion, cell death and mitochondrial dynamics. In addition to calcium, lipid homeostasis is also regulated at MERCs. MFN2 is important for the transfer of phosphatidylserine (PS) from the ER to mitochondria, where it is converted to phosphatidylethanolammine (PE), while the MAM-resident protein ACAT1 converts cholesterol to ceramide, a sphingolipid often abundantly found in cancer. Impaired lipid homeostasis has been associated with different kinds of tumors, such as liver, breast, pancreatic and colon cancers. For abbreviations, please refer to the text. This figure was created using images from Servier Medical Art (http://smart.servier.com). Servier Medical Art by Servier is licensed under a Creative Commons Attribution 3.0 Unported License.

Mitochondria–ER contacts (MERCs), biochemically isolated as the mitochondria-associated membranes (MAMs), were discovered on electron micrographs in the early 1950s [19,20]. However, MERCs’ importance in cell homeostasis was recognized only many years later, when regulation of phospholipid transfer and Ca^2+^ exchange was demonstrated [21,22]. Every cell has a specific number, length and thickness of MERCs [23]. In recent years, new tools have been developed to study MERCs number and length using confocal microscopy [24]. The architecture of the structural scaffold of MERCs is depicted in Figure 1, and is constituted by proteins that are inserted in the outer mitochondrial membrane (Protein tyrosine phosphatase interacting protein 51 (PTPIP51), Voltage-dependent anion channel 1 (VDAC1), Mitochondrial fission 1 (FIS1), Mitofusin 2 (MFN2), Pyruvate dehydrogenase kinase 4 (PDK4), Transglutaminase 2 (TG2) and ATPase family AAA domain containing 3A (ATAD3A)) and are able to interact with proteins residing in the ER membrane (VAMP-associated Protein B (VAPB), Inositol 1,4,5-triphosphate receptors (IP3R), Glucose-regulated protein 75 (GRP75), B-cell receptor-associated protein 31 (BAP31), Mitofusin 2 (MFN2), Motile sperm domain containing 2 (MOSPD2) and Binding immunoglobulin protein (BIP)) [8,25].

The close relationship brings advantages to both organelles. For example, protein folding that takes place in the ER relies on mitochondrial ATP, while ER-released Ca^2+^ that enters mitochondria positively regulates ATP synthesis. Metabolite flux also takes place at these MERCs, which are located in the MAMs [26]. Today, it is clear that MERCs represent hubs for signaling that control mitochondrial biology related to several aspects of the many functions of healthy cells, but also play a crucial role in pathologic conditions. In fact, MERCs control redox signaling, inflammation, autophagy and mitochondrial fission. Altered MERCs can deregulate Ca^2+^ homeostasis, phospholipid metabolism, mitochondrial morphology and dynamics [14,27]. MERCs can be considered as sensors of cell health. In fact, they can modulate cell cycle progression by sensing if the Ca^2+^ dynamic is regular and coordinated. Furthermore, if MERCs undergo transient dissociation, the ER stress response is induced, and then the ribosomes’ super-complexes transduce a signal to the ER and to some cytosolic partners, leading to the blocking of protein translation, thus arresting cell growth. Finally, the apoptotic pathway is the final stage of this process if ER dysfunction becomes permanent [28,29].

MERCs are particularly important in Ca^2+^ signaling (Figure 1) because they connect two of the most important organelles involved in Ca^2+^ homeostasis. In 1998, contact sites were identified as players in calcium (Ca^2+^) shuttling between mitochondria and ER [22]. Ca^2+^ is an important signaling ion in cell biology, and multiple cellular responses are evoked by local and/or transient changes in its concentration. Ca^2+^ is a relevant messenger for all cell compartments, but its concentration has to be precisely regulated, especially in the cytosol, where basal [Ca^2+^] is constantly reduced to nM concentration (≈100 nM) by the continuous activity of Ca^2+^ ATPases, which move Ca^2+^ into stores (ER, mitochondria) or the extracellular matrix (≈2 mM [Ca^2+^]). The cytosolic basal Ca^2+^ level can be rapidly altered upon activation of Ca^2+^ signaling. Indeed, it can almost instantly increase by up to 3 μM due to several selective Ca^2+^ channels located in the plasma membrane or on the ER surface [30]. Ca^2+^ is a highly reactive species because it can coordinate both charged and uncharged oxygen atoms in carbonyl groups of glutamate, aspartate, glutamine and asparagine. The interaction of calcium with the amino acids in turn can affect the properties and activities of a wide range of proteins and enzymes. For example, calmodulin (CaM), a 15 KDa protein with calcium-binding sites, undergoes substantial conformational changes, revealing internal hydrophobic patches on its surface, enabling interaction with some target proteins [31].

Several oncogenic pathways supporting tumor cell growth and survival depend on cellular metabolism and Ca^2+^ signaling [32]. Emerging data suggest that ER and mitochondria can reciprocally transduce signaling relevant for oncogenesis by MERCs. An increasing number of studies have revealed a link between the proliferation of cancer cells and MERCs’ functions, related to cell survival, adhesion, motility, invasion, metastasis and apoptotic resistance [33,34,35]. ER can load Ca^2+^ into mitochondria, offering new insights into mitochondria regulation and functions [36]. Multiple effects can be generated by Ca^2+^ dynamics in mitochondria, as follows: mitochondrial [Ca^2+^] can be taken up by the mitochondrial calcium uniporter MCU [37,38], regulating ATP production or the release of caspase cofactors [39]. The Krebs cycle enzymes α-ketoglutarate dehydrogenase and isocitrate dehydrogenase, as well as pyruvate dehydrogenase, are all Ca^2+^-dependent enzymes [40], thus also a mild increase in mitochondrial [Ca^2+^] results in elevated generation of ATP [41]. However, Ca^2+^ signals have a dual effect: excessive and prolonged [Ca^2+^] within the mitochondrial matrix can induce apoptosis, for example by triggering permeability transition pore (PTP) opening [42], a Ca^2+^-dependent channel [43], proposed to be formed by dimers of F_0_F_1_ ATP synthase [44]. Mitochondrial Ca^2+^ imbalance also induces the oligomerization of Bcl-2-associated X protein (BAX), so as to increase the permeability of mitochondrial membranes [45]. This process may lead to activation of the intrinsic apoptotic pathway and caspase activation by releasing cytochrome c, Smac/DIABLO and apoptosis-inducing factor (AIF) [46]. Several oncoproteins can interact at the MAMs with the inositol 1,4,5-triphosphate receptor (IP3R) responsible for Ca^2+^ release from the ER, and can thus modulate Ca^2+^ fluxes to the mitochondria, with consequent regulation of the induction of the intrinsic apoptotic pathway. Recently, it has been demonstrated that all three IP3R isoforms are required for maintaining MERCs [47], and that IP3R can bind with proteins like the phosphatase and tensin homolog deleted on chromosome 10 (PTEN), breast cancer type 1 susceptibility protein (BRCA1) and Bcl-2 [48]. PTEN, often mutated or absent in several cancers, can physical interact with IP3R3, thus favoring Ca^2+^ transfer by dephosphorylating both IP3R3 and AKT and leading to cell death [49]. On the contrary, IP3R phosphorylation by AKT reduces ER–mitochondria Ca^2+^ relocation and inhibits apoptosis in cancer cells [50,51,52]. Similarly to PTEN, BRCA1 can interact with IP3R1, supporting Ca^2+^ release and apoptosis [53]. Conversely, Bcl-2 binds IP3R isoforms, blocking Ca^2+^ transfer and conferring resistance to cell death. In fact, Bcl-2 overexpression is a key event for cancer cell survival and chemoresistance, as observed for the cisplatin treatment in ovarian cancer [54,55]. Recently, Transglutaminase type 2 (TG2) has also been shown to modulate Ca^2+^ fluxes and cell death by interacting with the glucose-regulated protein 75 (GRP75). GRP75 is one of the key proteins localized at MAMs, and is important for Ca^2+^ signaling, able to form a complex with IP3R and the voltage-dependent anion channel (VDAC). It has been demonstrated that GRP75 creates a molecular bridge between IP3R and VDAC1 at the MAMs, supporting Ca^2+^ transfer from ER to mitochondria [56]. A lack of TG2 leads to impaired Ca^2+^ movement from ER to mitochondria, and to a decreased number of MERCs controlling the interaction between IP3R and GRP75 at the MAMs [57]. TG2 overexpression promotes cell attachment, invasion and survival in breast cancer cells [58].

Ca^2+^ fluxes from ER to mitochondria are also tuned by Sigma receptor 1 (SIG1R), a chaperone located in different districts within the cell and enriched at MAMs, where it can interact with GRP78 (also called BIP) [59]. SIGR1 is overexpressed in cancer cells with high metastatic potential, where it can form a molecular complex with the small-conductance Ca^2+^-activated potassium (SKCa) channel and the Ca^2+^-release-activated Ca^2+^ modulator 1 (ORAI1), which favors cancer cells migration [60,61]. Whether such interaction also takes place at the level of MAMs involving the mitochondrial counterpart of the SKCa [62] remains to be determined. Finally, the oncoprotein Ras, a small GTPase that localizes at the plasma membrane–ER interface as well as at MAMs, prevents apoptotic cell death by modulating ER mitochondrial Ca^2+^ signaling [35]. Very recently, the UPR (unfolded protein response) signal transducer IRE1α has also been shown to act as a scaffold at MAMs, influencing Ca^2+^ signal and mitochondrial bioenergetics [63]. In addition to calcium signaling, redox signaling at MAMs is also emerging as a critical player in setting the cells’ behavior. Basal redox signaling occurs at MAMs and mediates cell growth and differentiation. Excessive ROS production causes oxidative stress, and by disrupting ER Ca^2+^ homeostasis may affect cancer progression. For example, superoxide anions can target IP3R thiol groups so as to affect MERCs and to modulate Ca^2+^ signaling by promoting Ca^2+^ transfer from the ER to mitochondria [64]. Such a cross-talk between redox and calcium signaling at the MERCs might turn out to be of great relevance for fighting different pathologies, ranging from neurodegeneration to cancer and diabetes.

Since mitochondria have several distinct functions, they can constantly change their shape to satisfy the cell requirements and modulate their functions. Thus, both the balance between oxidative and glycolytic metabolisms as well as the balance between fusion and fission, which is intimately linked to the bioenergetic efficiency of this organelle [46,65], are continuously tuned. Ca^2+^ modulates these structural changes both in normal and in cancer cells [66,67]—indeed, mitochondrial Ca^2+^ overload increases fragmentation. Ca^2+^ influx into the mitochondria has important consequences, not only in the context of apoptosis, but also concerning mitochondrial dynamics. For example, Ca^2+^ influx through VDAC and MCU induces rapid mitochondrial fission by phosphorylation of the dynamin-related protein 1 (DRP1) in neurons [68].

**Figure 2:**
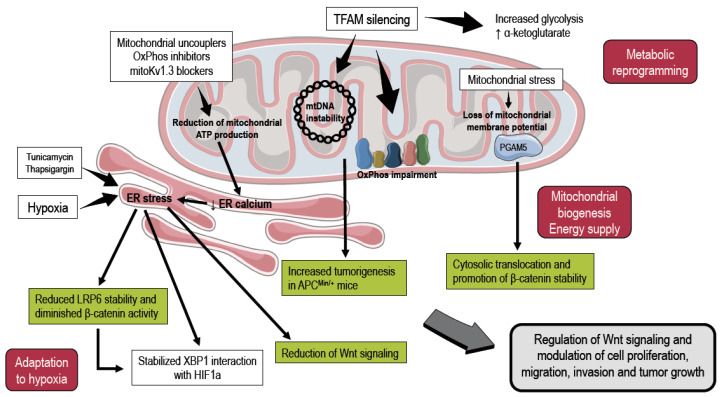
Interplay between mitochondria and Wnt signaling. Mitochondria can modulate intracellular signaling in different ways. ER stress induced by hypoxia or reduced ATP production in mitochondria leads to a decrease in β-catenin stability and a subsequent reduction in Wnt signaling. On the other hand, the loss of mitochondrial membrane potential can induce the translocation of PGAM5 into the cytosol, where it promotes β-catenin stability. TFAM, a mitochondrial transcription factor, is also thought to contribute to the complex regulation of Wnt signaling by mitochondria. All these events are important factors that allow a dynamic regulation of intracellular signaling. For further details, please refer to the text. This figure was created using images from Servier Medical Art (http://smart.servier.com). Servier Medical Art by Servier is licensed under a Creative Commons Attribution 3.0 Unported License.

It is not only ER that can modulate mitochondria through MERCs; mitochondria can also alter ER functionality via contact sites in healthy cells and under pathological conditions. Mitochondria can sustain the ER-located SERCA machinery by providing ATP for its function, thus preventing ER stress, and can also support lipid synthesis and the exchange of metabolites between the organelles [3,69,70]. MERCs allow both Ca^2+^ channeling and ATP translocation to the ER Ca^2+^ ATPase (SERCA), generating an efficient synergistic cycle. Mitochondrial membrane depolarization triggered by high Ca^2+^ levels is followed by a decrease in ATP synthesis, leading to decreased SERCA function, and ultimately leading to cell death [71]. The recent discovery of a mitochondria/ER-Wnt Axis [3] also provides evidence regarding the consequence of destroying this homeostatic mechanism [72,73]. Indeed, mitochondrial dysfunction leading to reduced ATP production and/or the disruption of mitochondria/ER junctions induces ER stress and affects the re-loading of Ca^2+^ into the ER, leading to a catastrophic and cyclic impairment of cell functions (Figure 2). Thus, a beneficial and synergistic Ca^2+^/ATP exchange between mitochondria and ER sustains cell life and cell proliferation in normal cells [3].

In addition to proliferative functions, Ca^2+^ signaling at the ER/mitochondria interface controls cell migration [74]. As examples, the ER resident STIM1 in the human umbilical vein acts locally on adhesion by enhancing Ca^2+^ influx and reloading ER Ca^2+^ stores in the front of the cell, so as to permit local Ca^2+^ pulses [75]. At the level of mitochondria, the upregulation of MCU activity was shown to significantly improve cell migration in vitro in MCF-7 tumor cells, as well as in vivo [76]. In accordance with this, the MCU channel is often found to be overexpressed in breast cancer biopsies of patients with tumor metastasis [76,77]. Whether the calcium flux among the two organelles ensured by MERCs plays a role in migration is still unclarified.

In addition to Ca^2+^ signaling, MAMs are also hubs for lipid metabolism (Figure 1), which is reprogrammed, as postulated by Otto Warburg, during oncogenic transformation [78]. In fact, the survival and proliferation of cancer cells is highly demanding in terms of energy, being thus influenced by de novo fatty acid synthesis [79]. In this scenario, MAMs have an important role in cholesterol, ceramide and phospholipids biosynthesis [80]. For example, Mitofusin-2, a protein essential for mitochondrial fusion and resident in the MAMs, can bind phosphatidylserine during its transport from the ER to the mitochondria, where phosphatidylserine is decarboxylated to produce phosphatidylethanolamine (Figure 1) [81]. In several cancer cells, compared to normal ones, Mitofusin-2 expression and the transfer of phosphatidylserine are reduced, causing decreased phospholipid synthesis. In particular, the downregulation of Mitofusin-2 in hepatic cells leads to non-alcoholic steatohepatitis, which finally induces liver cancer [82]. Not only biosynthesis, but also lipid degradation, is important and impaired in several types of cancer, and the enzymes involved in this pathways have been specifically found at the MAMs [48]. Lipids are degraded to triacylglycerols and cholesteryl esters that are incorporated into lipid droplets, which are increased in number in glioblastoma [83], leukemia [84], breast [85], pancreatic [86] and colon cancers [87]. Furthermore, due to the activity of the enzyme localized at the MAMs acyl-coenzyme A: cholesterol acyltransferase-1 (ACAT1) [88], cholesterol is converted into ceramide and accumulated in lipid droplets (Figure 1). Ceramide is more present in lipid droplets in cancer cells than in healthy ones, and this increase is correlated with cancer cell proliferation and metastatic potential, as well as with poor prognosis in pancreatic and prostate cancers [89,90]. Ceramide accumulation due to ACAT1 activity has also been related to activation of the phosphatidylinositol-3-kinase (PI3K)/AKT, and the mammalian target of rapamycin (mTOR) or the caveolin-1/mitogen-activated protein kinase (MAPK) signaling pathways [91,92,93].

The modulation of the length of the ER/mitochondria contact surface as well as of the distance between the two organelles impacts both on the function of the involved organelles as well as on cellular signaling. As an example, a recent work shows that pharmacological inhibition of the mitochondrial pyruvate dehydrogenase kinase 4 (PDK4) activity, which suppresses the conversion of pyruvate to acetyl CoA via inhibitory phosphorylation of the pyruvate dehydrogenase complex, dampens MAMs formation and improves insulin signaling by preventing MAM-induced mitochondrial Ca^2+^ accumulation, mitochondrial dysfunction and ER stress. Indeed, *Pdk4*^-/-^ mice exhibited reduced MAMs formation and were protected against diet-induced skeletal muscle insulin resistance [94]. Importantly, the length of the MERCs can be modulated by diet, as it was shown to increase in vivo in the postprandial liver [95]. On the contrary, the MAMs integrity was shown to be reduced in the liver after feeding [96]. Reduction in MERCs number, beginning three days after the introduction of a high-fat diet, was observed in another study [97]. In addition, recent evidence indicates that MAMs could be a hub of hepatic insulin signaling and nutrient sensing. The view is emerging that the dynamic regulation of MERCs/MAMs affects mitochondrial physiology and the adaptation of cellular metabolism to nutrient availability, and that chronic MAMs disruption participates in the metabolic inflexibility associated with metabolic disorders [98]. Thus, diet can indeed modulate MAMs that in turn seem to affect ER stress. ER stress, hypoxia and starvation all induce tighter contacts, while contacts are looser with high glucose levels [13].

Finally, MERCs dysfunction-linked ER stress impacts on several important cellular processes, including Wnt signaling [99,100], which is one of the most important signaling pathways determining either cell life or death (Figure 2). Canonical Wnt signaling relies on the progressive accumulation of unphosphorylated β-catenin in the cytoplasm, and its translocation into the nucleus to act as a transcriptional co-activator of a plethora of transcription factors, including the T cell factor/lymphoid enhancer factor (TCF/LEF) family. In the absence of Wnt ligands, β-catenin is degraded by the destruction complex that includes Axin, adenomatosis polyposis coli (APC), protein phosphatase 2A (PP2A), glycogen synthase kinase 3 β (GSK3β) and casein kinase 1α (CK1 α). Wnt initiates signaling events by binding to a receptor complex. Subsequently, the cytoplasmic adaptor protein Dishevelled (Dvl) is phosphorylated and inhibits GSK3β activity through its association with Axin, leading to the accumulation of unphosphorylated β-catenin [101]. Wnt/β-catenin signaling is often constitutively activated in cancer cells (e.g., [102]), including breast [103] and colon cancer [104], thereby actively participating in conferring unlimited proliferative potential to these pathologic cells. The aberrant activation of Wnt/β-catenin signaling promotes the development of several cancers [105]; indeed, Wnt/β-catenin signaling is often constitutively activated in cancer cells, thereby actively participating in conferring unlimited proliferative potential to these pathologic cells, and leading to a worsened outcome in patients in whom this pathway is elevated [58]. Wnt has also been shown to be involved in the EMT that drives cells towards differentiation into migratory mesenchymal and invasive cells, able to form metastasis [106]. In this context, following mitochondrial stress due to the loss of mitochondrial membrane potential, mitochondria can modulate β-catenin by favoring the translocation in the cytosol of PGAM5, a serine/threonine phosphatase that resides in the inner mitochondrial membrane and that interacts with AXIN1-promoting β-catenin de-phosphorylation and stabilization (Figure 2) [107]. This signaling pathway support mitochondrial biogenesis, replacing damaged mitochondria with healthy ones, in turn supplying energy necessary for cancer cell progression [108]. Furthermore, Wnt signaling has been recently shown to be modulated also by mitochondrial retrograde signals [72,109]. Thus, mitochondrial DNA (mtDNA) instability leads to increased tumorigenesis by enhancing ROS production in TFAM^+/-^ (Transcription factor A mitochondria) heterozygous knock-out mice crossed with adenomatous polyposis coli multiple intestinal neoplasia (APC^Min/+^) mouse cancer models [109]. Similarly to the mitochondria/ER-Wnt axis, the silencing of TFAM causes the loss of mtDNA, a deficiency in oxidative phosphorylation and a decrease in Wnt/β-catenin target genes, impairing the ability of colon cancer cells to form tumors in vitro as well as in vivo [72] (Figure 2). Furthermore, TFAM depletion leads to a cellular metabolic reprogramming by enhancing glycolysis and increasing production of α-ketoglutarate, which in turn can downregulate Wnt signaling by the reduction of hypoxia-inducible factor 1α (Hif1α expression [72]). Moreover, Hif1α has also been shown to support colon cancer cell proliferation by interacting with spliced X-box-binding protein 1 (XBP1). Indeed, during hypoxia, induced ER stress reduces low-density lipoprotein receptor-related protein 6 (LRP6), which in turns diminishes β-catenin expression [110] (Figure 2).

Thus, the molecules/processes involved in this signaling pathway are considered attractive targets for the design of new chemotherapeutic agents [106]. To date, some of the small molecules inhibiting Wnt signaling have entered clinical testing [111]. In this scenario, the direct mitochondrial modulation of Wnt signaling is a new frontier in oncology [73]. Indeed, mitochondrial uncouplers, like nonactin (a member of a family of naturally occurring cyclic ionophores known as macrotetrolide antibiotics), have been shown to selectively kill mutant β-catenin-harboring tumor cells in vivo in xenograft mouse models [112]. Furthermore, pyrvinium pamoate, an FDA-approved drug that inhibits NADH-fumarate reductase systems leading to electron transport chain and ATP synthesis decrease [113], was demonstrated to block Wnt/β-catenin signaling by selectively potentiating casein kinase 1α (CK1α) in colorectal cancer cells harboring APC mutation and aberrant Wnt activation [114]. Recently, a cationic cyclometalated platinum(ii) complex has been shown to selectively accumulate in the mitochondria and to disrupt them, thus reducing the proliferation and migration of cancer cells, and finally blocking Wnt signaling by preventing β-catenin translocation into the nucleus [115]. Finally, metformin, a known inhibitor of mitochondrial respiratory complex I, impairs colorectal cancer cell growth by inhibiting the Wnt/β-catenin signaling pathway [116]. Thus, growing evidence points to a modulation of this important signaling pathway by mitochondrial fitness.

## 3. Mitochondria and Lysosomes

A peculiar characteristic of cancer cells is that of being able to indefinitely expand. However, this uncontrolled growth is accompanied by a rapid depletion of cellular nutrients, and an accumulation of aggregated proteins and damaged organelles. To overcome cellular stress and to guarantee a high level of available biomolecules, the building blocks necessary to sustain cell growth, cancer cells reprogram their metabolism and upregulate lysosomal biogenesis and the expression of lysosomal enzymes. Indeed, as the waste disposal system of the cell, lysosomal dysfunction plays a key role in cancer development. Using ATP hydrolysis-derived energy, lysosomal vATPase pumps protons against the electrochemical gradient from the cytosol into the organelle, acidifying the lysosomal lumen and allowing the correct function of the acidic lysosomal hydrolases that catalyze macromolecule breakdown [117]. Moreover, lysosomes mediate autophagy, the mechanism that permits the digestion of intracellular material (either damaged or during starvation), to sustain cell survival and proliferation. While damaged mitochondria trigger cell death, lysosome-mediated mitophagy plays a cytoprotective role favoring tumorigenesis. On the other hand, damaged lysosomes can release proteolytic enzymes into the cytosol, promoting apoptotic process [118]. For that reason, cancer cells have developed many strategies to overcome the lysosomal death pathway. For example, cancer cells overexpress phosphatidylinositol 3-kinase (PI3K) and the Heat shock protein 70 (Hsp70) to stabilize the lysosomal membrane and to prevent its rupture [119,120,121].

Lysosome membranes are the site of action of target of rapamycin (mTOR), a protein kinase involved in several biological process, whose mutations can trigger carcinogenesis [122]. mTOR is a key component of several complexes, including mTOR complex 1 (mTORC1), mTOR complex 2 (mTORC2) and mTOR complex 3 (mTORC3). mTORC1 regulates cell proliferation, and monitors nutrients and energy accessibility to maintain metabolic homeostasis. Thus, de-regulation of the mTOR signaling pathway is observed in many cancers, among them pancreatic, renal, breast, liver, prostate, and lung carcinomas. The upregulation of mTOR signaling suppresses autophagy, enhancing cellular stress, which in turn can promote tumor growth, metabolic alterations, angiogenesis and metastasis formation [123].

To allow the proper performance of lysosomes, several ion channels finely adjust ion homeostasis within the organelle [124]. The fundamental cancer cell functions, such as cell survival, proliferation and migration, are maintained by ion fluxes across the membranes, and in particular Ca^2+^ plays a crucial role. While the concentration of Ca^2+^ within the cell in the cytosol is about 100 nM, in the lysosome lumen it is about 500 μM [125]. Ca^2+^ fluxes across lysosomal membranes are regulated by several ion channels, like those of the TRP family, namely TRPM2, TRPML1, TRPML2 and TRPML3, and those of the TPC family [124]. Upon activation by small molecules, synthetic compounds or nutrient starvation, these channels guide Ca^2+^ and other cations from the lumen of the lysosome into the cytosol. The increased cytosolic Ca^2+^ concentration leads to the dephosphorylation and activation of TFEB, the master regulator of lysosome biogenesis and autophagy-related genes, inducing the removal of damaged cellular components and re-establishing organelle homeostasis [126]. Lysosomal calcium release through TRPML1 binds calmodulin, favoring its association with mTOR and finally activating mTORC1 [127]. Moreover, some evidence reveals that TPC channels directly interact with mTOR, acting as nutrient sensors within the cell, and are also required for the uptake and processing of proteins in the endosome. In addition, Ca^2+^ can activate the lysosomal big-conductance K^+^ (BK) channels, known to be implicated in cancer migration, proliferation and metastasis [128]. BK can couple with TRPML1 channels, forming a complex within the lysosomal membrane. Together, BK and TRPML1 channels facilitate the efflux of Ca^2+^ from lysosomes, which mediates lysosomal membrane trafficking. Improper conductance through these lysosomal ion channels has been associated with many diseases; in particular, BK and TPC have been linked to cancer cell proliferation and metastasis formation [124,128].

During carcinogenesis, mitochondria also adjust their ion homeostasis to favor cellular growth and metabolism [129]. An increased mitochondrial biogenesis is important for cancer cells in order to sustain cellular biosynthetic and respiratory capacity, upregulating mitochondrial metabolism to support redox balance and to obtain sufficient energy to enhance cell growth [130]. Mitochondrial biogenesis and bioenergetics are modulated by mTOR (the mammalian target of rapamycin) in a transcription-dependent and -independent manner, modulating PGC-1α activity [131], the master regulator of mitochondrial biogenesis. During carcinogenesis, autophagy, particularly mitophagy, also plays an important role because its regulation can promote both pro- and anti-tumorigenic processes in relation to the tumor stage. In some stages, decreased mitophagy permits the permanence of damaged mitochondria, which increases tumor-promoting ROS production. On the contrary, established tumors need mitophagy to overcome stress and survive. Many studies show also an imbalance between fission and fusion in the mitochondria of cancer cells, with the predominance of a fragmented mitochondrial network in tumor cells [129].

**Figure 3:**
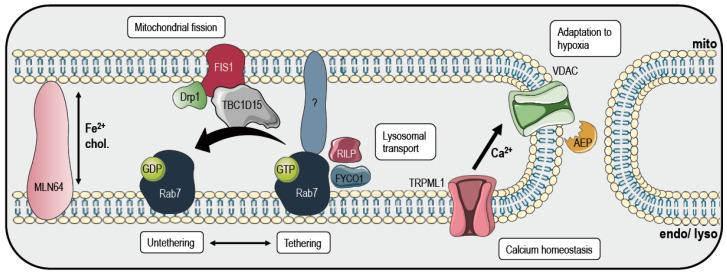
Signaling at mitochondria–lysosome contact sites. GTP-bound Rab7 is important for the tethering of the two organelles and also acts as a docking site for FYCO1 and RILP, which are responsible for the anterograde and retrograde transport of lysosomes among microtubules. The GTP-ase activating protein TBC1D15 is recruited by FIS1 and promotes GTP hydrolysis, leading to an untethering of mitochondria and lysosomes. FIS1 also recruits Drp1, important for mitochondrial fission. MLN64, a presumptive tether protein, contributes to the exchange of iron and cholesterol (chol.). Microfusions of the membranes during hypoxia bring the asparagine endopeptidase (AEP) into close proximity with VDAC, which could protect mitochondria from mitophagy and increase survival by cleaving the C-terminus of the channel. Finally, TRPML1 releases calcium from lysosomes at mitochondria–lysosome contact sites. This figure was created using images from Servier Medical Art (http://smart.servier.com). Servier Medical Art by Servier is licensed under a Creative Commons Attribution 3.0 Unported License.

Considering the intertwined tasks between lysosomes and mitochondria, the question of how these two organelles can communicate with each other became an important issue. Nowadays, some rather limited information about the interactions between endosomes or lysosomes and mitochondria is available (Figure 3). Lysosomes and mitochondria directly interact during mitophagy or the degradation of mitochondrial-derived vesicles. However, they also interact in normal conditions, but this novel field needs to be further explored [132]. Membrane contacts are maintained by tethering proteins, which physically bridge organelles’ membranes. Additional proteins are involved in the regulation of these contacts’ function, which mediate metabolite trafficking among the organelles and their responses to environment [133]. In the case of the lysosome–mitochondria tethering process, the small GTPase Rab7 is the main regulator of their dynamics, Rab7 being the master regulator of lysosomal dynamics (Figure 3). It is present in the cytoplasm in the inactive GDP binding state, while GTP binding promotes its lysosomal localization and activation. Once attached to lysosomal membranes, other effector proteins bind Rab7 in the GTP binding state [134]. Among the Rab7 effector proteins, TBC1D15 is a GTP-ase activating protein. It is recruited by the mitochondrial Fis1, and after the binding of Rab7, it promotes the hydrolysis to a GDP-bound state, and mitochondria–lysosomes untether [135]. FYCO1 [136] and RILP [137] bind GTP-bound Rab7 to respectively promote anterograde and retrograde transports among microtubules. Moreover, mitochondria–lysosomes contact sites mark the areas of mitochondrial fission, as observed by the presence of Drp1 [134]. Drp1 is a dynamin-related GTPase that, once activated, oligomerizes around the OMM, binds its adaptors, like Fis1, constricting and dividing the mitochondria into two separate organelles [138]. Thus, mitochondria–lysosome contact sites also regulate mitochondrial dynamics. Another function of mitochondria–lysosome tethering is the favoring of the flux of metabolites, lipids, iron and ions among them. The endosomal protein MLN64 seems to be necessary for the physical coupling between endosome and mitochondria, supporting the transfer of cholesterol and iron between these two organelles (Figure 3). Furthermore, oxidative stress seems to enhance association between endosomes and mitochondria, decreasing apoptosis and enhancing the exchange of metabolites and lipids among the organelles, while favoring the repair of damaged mitochondria [65]. Finally, recent evidence indicates that during hypoxia, local microfusions between mitochondria and endolysosomes can favor the post-translational C-terminus cleavage of VDAC1, a voltage-dependent anion channel that moves ions, including calcium, and metabolites from and into mitochondria. The cleavage is mediated by the endolysosomal asparagine endopeptidase, marking mitochondria for protection from mitophagy to promote cell survival during hypoxia [139].

The contact sites between lysosomes and mitochondria seem to play a crucial role in calcium homeostasis as well. In particular, lysosomal calcium released by TRPML1 has been recently shown to promote calcium transfer to mitochondria, which was mediated by the tethering of mitochondria–lysosome contact sites (Figure 3). Interestingly, the disruption of this contact and calcium transfer has been linked to mutations of TRPML1, and to the lysosomal storage disorder Mucolipidosis type IV [140].

## 4. Mitochondria and Peroxisomes

Peroxisomes are single membrane-surrounded dynamic organelles that play important roles in biosynthetic processes and signal transduction, since they are involved in phospholipid biosynthesis, fatty acid α- and β-oxidation, bile acid and docosahexaenoic acid synthesis, glyoxylate metabolism, amino acid catabolism, polyamine oxidation, ROS and nitrogen species metabolism, inflammation and innate immunity [141,142]. Peroxisomes can cooperate with mitochondria to achieve fatty acid β-oxidation and to maintain ROS balance [143]. The β-oxidation of fatty acids is executed both in the mitochondria and peroxisomes by four consecutive reactions [144]. The β-oxidation of some lipids is initiated in the peroxisomes and completed in the mitochondria, where intermediates are used to produce energy [144].

**Figure 4:**
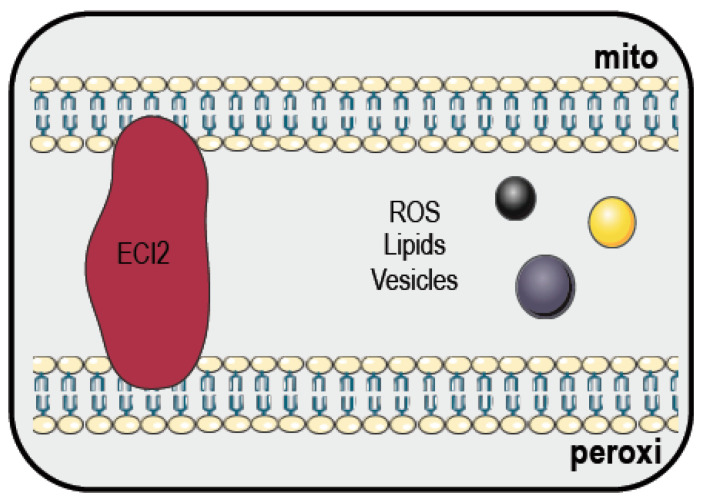
Mitochondria–peroxisome contact sites. At these contact sites, enoyl-CoA isomerase 2 (ECI2) is the only physical tethering complex identified to date. The two organelles are thought to communicate with each other also through vesicles, ROS and lipids. Indeed, peroxisomes are important for ROS clearance and β-oxidation of fatty acids. Please refer to the text for further details. This figure was created using images from Servier Medical Art (http://smart.servier.com). Servier Medical Art by Servier is licensed under a Creative Commons Attribution 3.0 Unported License.

In mammalian cells, the contact sites among mitochondria and peroxisomes are mediated by a complex constituted by a splice variant of enoyl-CoA isomerase 2 (ECI2) [145] (Figure 4). Mitochondria and peroxisomes interplay could be supported not only by direct membrane contact sites, but also through vesicle transport and signaling molecules [146]. Importantly, peroxisomes are essential in preserving mitochondrial functions, and their dysfunction impairs mitochondrial metabolism, morphology and biosynthesis, and is linked to several disease, among which is cancer [145].

Furthermore, peroxisomes are important organelles involved in the clearance of ROS, since they contain catalase to break H_2_O_2_. The impairment of catalase activity will lead to mitochondrial oxidative stress response induction, which could trigger damages to the cell components [147,148]. ROS, such as H_2_O_2_, are important signaling molecules able to support cancer proliferation by, for example, activating the epidermal growth factor (EGF) of the platelet-derived growth factor (PDGF), or by regulating the PTEN activity of the phosphoinositide 3-kinase (PI3K) signaling pathway [149,150]. In addition, ROS can promote tumor cell proliferation during hypoxia by preventing hypoxia inducible factors (HIFs) degradation by prolyl hydroxylases (PHDs) [149]. Generally, tumor cells display a higher ROS production, which favors DNA damage and increased mutation rates, leading to a malignant phenotype [151]. Moreover, ROS support also the angiogenesis, invasion and migration of transformed cancer cells [152]. Whether and how mitochondria–peroxisome contact sites contribute to the setting of cellular ROS level is still unclear, to the best of our knowledge.

## 5. Conclusions

In summary, while the relevance of the targeting of mitochondrial metabolism by molecules able to specifically disrupt mitochondrial fitness and trigger cell death exclusively in cancer cells has been recognized by the scientific community as a promising strategy against cancer [153], the exploitation of the results regarding inter-organellar contact sites for pharmacological intervention is still limited. In this context, the recent discovery of two drugs able to modulate both mitochondrial functions and, at sub-lethal concentrations, Wnt signaling, by affecting the functional coupling between mitochondria and the ER, might be a promising example. The same drugs at higher concentrations trigger mitochondrial ROS production by the direct targeting of a mitochondrial potassium channel that is overexpressed in cancer cells, and induce apoptosis selectively in cancer cells, even in vivo, in melanoma and pancreatic ductal adenocarcinoma models [154]. Similarly to MAMs, the contact sites between mitochondria and lysosomes, as well as peroxisomes, are emerging as important contributors to correct calcium and ion homeostasis. Future work is required to fully explore the complexity of the machineries responsible for the tethering of these organelles, as well as for the identification of drugs able to specifically modulate ion/metabolite fluxes among the organelles, possibly leading to the selective elimination of cancer cells.

## References

[1] Dyall S.D., Brown M.T., Johnson P.J. (2004). Ancient Invasions: From Endosymbionts to Organelles. Science.

[2] Timmis J.N., Ayliff M.A., Huang C.Y., Martin W. (2004). Endosymbiotic gene transfer: Organelle genomes forge eukaryotic chromosomes. Nat. Rev. Genet..

[3] Costa R., Peruzzo R., Bachmann M., Montà G.D., Vicario M., Santinon G., Mattarei A., Moro E., Quintana-Cabrera R., Scorrano L. (2019). Impaired Mitochondrial ATP Production Downregulates Wnt Signaling via ER Stress Induction. Cell Rep..

[4] Bhola P.D., Letai A. (2016). Mitochondria-Judges and Executioners of Cell Death Sentences. Mol. Cell.

[5] Youle R.J. (2019). Mitochondria—Striking a balance between host and endosymbiont. Science.

[6] Merry T.L., Ristow M. (2016). Mitohormesis in exercise training. Free Radic. Biol. Med..

[7] Gomez-Suaga P., Paillusson S., Miller C.C.J. (2017). ER-mitochondria signaling regulates autophagy. Autophagy.

[8] Gordaliza-Alaguero I., Cantó C., Zorzano A. (2019). Metabolic implications of organelle–mitochondria communication. EMBO Rep..

[9] Gómez-Suaga P., Bravo-San Pedro J.M., González-Polo R.A., Fuentes J.M., Niso-Santano M. (2018). ER-mitochondria signaling in Parkinson’s disease review-article. Cell Death Dis..

[10] Naia L., Ferreira I.L., Ferreiro E., Rego A.C. (2017). Mitochondrial Ca^2+^ handling in Huntington’s and Alzheimer’s diseases—Role of ER-mitochondria crosstalk. Biochem. Biophys. Res. Commun..

[11] Basso V., Marchesan E., Peggion C., Chakraborty J., von Stockum S., Giacomello M., Ottolini D., Debattisti V., Caicci F., Tasca E. (2018). Regulation of ER-mitochondria contacts by Parkin via Mfn2. Pharmacol. Res..

[12] Rieusset J. (2017). Role of endoplasmic reticulum-mitochondria communication in type 2 diabetes. Advances in Experimental Medicine and Biology.

[13] Simmen T., Herrera-Cruz M.S. (2018). Plastic mitochondria-endoplasmic reticulum (ER) contacts use chaperones and tethers to mould their structure and signaling. Curr. Opin. Cell Biol..

[14] Morciano G., Marchi S., Morganti C., Sbano L., Bittremieux M., Kerkhofs M., Corricelli M., Danese A., Karkucinska-Wieckowska A., Wieckowski M.R. (2018). Role of Mitochondria-Associated ER Membranes in Calcium Regulation in Cancer-Specific Settings. Neoplasia.

[15] Xia M.F., Zhang Y.Z., Jin K., Lu Z.T., Zeng Z., Xiong W. (2019). Communication between mitochondria and other organelles: A brand-new perspective on mitochondria in cancer. Cell Biosci..

[16] Eisenhut M., Hocken N., Weber A.P.M. (2015). Plastidial metabolite transporters integrate photorespiration with carbon, nitrogen, and sulfur metabolism. Cell Calcium.

[17] Valm A.M., Cohen S., Legant W.R., Melunis J., Hershberg U., Wait E., Cohen A.R., Davidson M.W., Betzig E., Lippincott-Schwartz J. (2017). Applying systems-level spectral imaging and analysis to reveal the organelle interactome. Nature.

[18] Ikonen E. (2008). Cellular cholesterol trafficking and compartmentalization. Nat. Rev. Mol. Cell Biol..

[19] Lever J.D., Chappell J.B. (1958). Mitochondria isolated from rat brown adipose tissue and liver. J. Biophys. Biochem. Cytol..

[20] Copeland D.E., Dalton A.J. (1959). An association between mitochondria and the endoplasmic reticulum in cells of the pseudobranch gland of a teleost. J. Biophys. Biochem. Cytol..

[21] Vance J.E. (1990). Phospholipid synthesis in a membrane fraction associated with mitochondria. J. Biol. Chem..

[22] Rizzuto R., Pinton P., Carrington W., Fay F.S., Fogarty K.E., Lifshitz L.M., Tuft R.A., Pozzan T. (1998). Close contacts with the endoplasmic reticulum as determinants of mitochondrial Ca^2+^ responses. Science.

[23] Giacomello M., Pellegrini L. (2016). The coming of age of the mitochondria-ER contact: A matter of thickness. Cell Death Differ..

[24] Calì T., Ottolini D., Vicario M., Catoni C., Vallese F., Cieri D., Barazzuol L., Brini M. (2019). splitGFP Technology Reveals Dose-Dependent ER-Mitochondria Interface Modulation by α-Synuclein A53T and A30P Mutants. Cells.

[25] Csordás G., Weaver D., Hajnóczky G. (2018). Endoplasmic Reticulum–Mitochondrial Contactology: Structure and Signaling Functions. Trends Cell Biol..

[26] Fan Y., Simmen T. (2019). Mechanistic Connections between Endoplasmic Reticulum (ER) Redox Control and Mitochondrial Metabolism. Cells.

[27] Doghman-Bouguerra M., Lalli E. (2019). ER-mitochondria interactions: Both strength and weakness within cancer cells. Biochim. Biophys. Acta Mol. Cell Res..

[28] Rozpedek W., Pytel D., Mucha B., Leszczynska H., Diehl J.A., Majsterek I. (2016). The Role of the PERK/eIF2α/ATF4/CHOP Signaling Pathway in Tumor Progression During Endoplasmic Reticulum Stress. Curr. Mol. Med..

[29] Savignac M., Simon M., Edir A., Guibbal L., Hovnanian A. (2014). SERCA2 dysfunction in darier disease causes endoplasmic reticulum stress and impaired cell-to-cell adhesion strength: Rescue by miglustat. J. Investig. Dermatol..

[30] Clapham D.E. (1995). Calcium Signaling. Cell.

[31] Stigler J., Rief M. (2012). Calcium-dependent folding of single calmodulin molecules. Proc. Natl. Acad. Sci. USA.

[32] Cardenas C., Pinton P., Bultynck G. (2018). Editorial: Inter-organelle calcium communication in cancer. Front. Oncol..

[33] Giorgi C., Bonora M., Sorrentino G., Missiroli S., Poletti F., Suski J.M., Ramirez F.G., Rizzuto R., Di Virgilio F., Zito E. (2015). P53 at the endoplasmic reticulum regulates apoptosis in a Ca^2+^-dependent manner. Proc. Natl. Acad. Sci. USA.

[34] Betz C., Stracka D., Prescianotto-Baschong C., Frieden M., Demaurex N., Hall M.N. (2013). MTOR complex 2-Akt signaling at mitochondria-associated endoplasmic reticulum membranes (MAM) regulates mitochondrial physiology. Proc. Natl. Acad. Sci. USA.

[35] Rimessi A., Marchi S., Patergnani S., Pinton P. (2013). H-Ras-driven tumoral maintenance is sustained through caveolin-1-dependent alterations in calcium signaling. Oncogene.

[36] Raffaello A., de Stefani D., Rizzuto R. (2012). The mitochondrial Ca^2+^ uniporter. Cell Calcium.

[37] De Stefani D., Raffaello A., Teardo E., Szabó I., Rizzuto R. (2011). A forty-kilodalton protein of the inner membrane is the mitochondrial calcium uniporter. Nature.

[38] Baughman J.M., Perocchi F., Girgis H.S., Plovanich M., Belcher-Timme C.A., Sancak Y., Bao X.R., Strittmatter L., Goldberger O., Bogorad R.L. (2011). Integrative genomics identifies MCU as an essential component of the mitochondrial calcium uniporter. Nature.

[39] De Stefani D., Rizzuto R., Pozzan T. (2016). Enjoy the Trip: Calcium in Mitochondria Back and Forth. Annu. Rev. Biochem..

[40] McCormack J.G., Denton R.M. (1993). Mitochondrial Ca^2+^ transport and the role of intramitochondrial Ca2+ in the regulation of energy metabolism. Dev. Neurosci..

[41] Hansford R.G., Zorov D. (1998). Role of mitochondrial calcium transport in the control of substrate oxidation. Mol. Cell. Biochem..

[42] Mammucari C., Gherardi G., Rizzuto R. (2017). Structure, activity regulation, and role of the mitochondrial calcium uniporter in health and disease. Front. Oncol..

[43] Das A.M., Harris D.A. (1990). Control of mitochondrial ATP synthase in heart cells: Inactive to active transitions caused by beating or positive inotropic agents. Cardiovasc. Res..

[44] Urbani A., Giorgio V., Carrer A., Franchin C., Arrigoni G., Jiko C., Abe K., Maeda S., Shinzawa-Itoh K., Bogers J.F.M. (2019). Purified F-ATP synthase forms a Ca^2+^-dependent high-conductance channel matching the mitochondrial permeability transition pore. Nat. Commun..

[45] Petronilli V., Penzo D., Scorrano L., Bernardi P., Di Lisa F. (2001). The mitochondrial permeability transition, release of cytochrome c and cell death. Correlation with the duration of pore openings in situ. J. Biol. Chem..

[46] Huang Q., Cao H., Zhan L., Sun X., Wang G., Li J., Guo X., Ren T., Wang Z., Lyu Y. (2017). Mitochondrial fission forms a positive feedback loop with cytosolic calcium signaling pathway to promote autophagy in hepatocellular carcinoma cells. Cancer Lett..

[47] Bartok A., Weaver D., Golenár T., Nichtova Z., Katona M., Bánsághi S., Alzayady K.J., Thomas V.K., Ando H., Mikoshiba K. (2019). IP3 receptor isoforms differently regulate ER-mitochondrial contacts and local calcium transfer. Nat. Commun..

[48] Simoes I.C.M., Morciano G., Lebiedzinska-Arciszewska M., Aguiari G., Pinton P., Potes Y., Wieckowski M.R. (2020). The mystery of mitochondria-ER contact sites in physiology and pathology: A cancer perspective. Biochim. Biophys. Acta Mol. Basis Dis..

[49] Bononi A., Bonora M., Marchi S., Missiroli S., Poletti F., Giorgi C., Pandolfi P.P., Pinton P. (2013). Identification of PTEN at the ER and MAMs and its regulation of Ca^2+^ signaling and apoptosis in a protein phosphatase-dependent manner. Cell Death Differ..

[50] Khan M.T., Wagner L., Yule D.I., Bhanumathy C., Joseph S.K. (2005). Akt kinase phosphorylation of inositol 1,4,5-trisphosphate receptors. J. Biol. Chem..

[51] Marchi S., Marinello M., Bononi A., Bonora M., Giorgi C., Rimessi A., Pinton P. (2012). Selective modulation of subtype III IP3R by Akt regulates ER Ca^2+^ release and apoptosis. Cell Death Dis..

[52] Szado T., Vanderheyden V., Parys J.B., De Smedt H., Rietdorf K., Kotelevets L., Chastre E., Khan F., Landegren U., Söderberg O. (2008). Phosphorylation of inositol 1,4,5-trisphosphate receptors by protein kinase B/Akt inhibits Ca^2+^ release and apoptosis. Proc. Natl. Acad. Sci. USA.

[53] Hedgepeth S.C., Garcia M.I., Wagner L.E., Rodriguez A.M., Chintapalli S.V., Snyder R.R., Hankins G.D.V., Henderson B.R., Brodie K.M., Yule D.I. (2015). The BRCA1 tumor suppressor binds to inositol 1,4,5-trisphosphate receptors to stimulate apoptotic calcium release. J. Biol. Chem..

[54] Monaco G., Decrock E., Akl H., Ponsaerts R., Vervliet T., Luyten T., De Maeyer M., Missiaen L., Distelhorst C.W., De Smedt H. (2011). Selective regulation of IP 3-receptor-mediated Ca^2+^ signaling and apoptosis by the BH4 domain of Bcl-2 versus Bcl-Xl. Cell Death Differ..

[55] Xu L., Xie Q., Qi L., Wang C., Xu N., Liu W., Yu Y., Li S., Xu Y. (2018). Bcl-2 overexpression reduces cisplatin cytotoxicity by decreasing ER-mitochondrial Ca^2+^ signaling in SKOV3 cells. Oncol. Rep..

[56] Szabadkai G., Bianchi K., Várnai P., De Stefani D., Wieckowski M.R., Cavagna D., Nagy A.I., Balla T., Rizzuto R. (2006). Chaperone-mediated coupling of endoplasmic reticulum and mitochondrial Ca^2+^ channels. J. Cell Biol..

[57] D’Eletto M., Rossin F., Occhigrossi L., Farrace M.G., Faccenda D., Desai R., Marchi S., Refolo G., Falasca L., Antonioli M. (2018). Transglutaminase Type 2 Regulates ER-Mitochondria Contact Sites by Interacting with GRP75. Cell Rep..

[58] Mangala L.S., Fok J.Y., Zorrilla-Calancha I.R., Verma A., Mehta K. (2007). Tissue transglutaminase expression promotes cell attachment, invasion and survival in breast cancer cells. Oncogene.

[59] Hayashi T., Su T.P. (2007). Sigma-1 Receptor Chaperones at the ER- Mitochondrion Interface Regulate Ca^2+^ Signaling and Cell Survival. Cell.

[60] Gueguinou M., Crottès D., Chantôme A., Rapetti-Mauss R., Potier-Cartereau M., Clarysse L., Girault A., Fourbon Y., Jézéquel P., Guérin-Charbonnel C. (2017). The SigmaR1 chaperone drives breast and colorectal cancer cell migration by tuning SK3-dependent Ca^2+^ homeostasis. Oncogene.

[61] Aydar E., Onganer P., Perrett R., Djamgoz M.B., Palmer C.P. (2006). The expression and functional characterization of sigma (σ) 1 receptors in breast cancer cell lines. Cancer Lett..

[62] Krabbendam I.E., Honrath B., Culmsee C., Dolga A.M. (2018). Mitochondrial Ca(2+)-activated K(+) channels and their role in cell life and death pathways. Cell Calcium.

[63] Carreras-Sureda A., Jaña F., Urra H., Durand S., Mortenson D.E., Sagredo A., Bustos G., Hazari Y., Ramos-Fernández E., Sassano M.L. (2019). Non-canonical function of IRE1α determines mitochondria-associated endoplasmic reticulum composition to control calcium transfer and bioenergetics. Nat. Cell Biol..

[64] Bánsághi S., Golenár T., Madesh M., Csordás G., RamachandraRao S., Sharma K., Yule D.I., Joseph S.K., Hajnóczky G. (2014). Isoform- and species-specific control of inositol 1,4,5-trisphosphate (IP3) receptors by reactive oxygen species. J. Biol. Chem..

[65] Giacomello M., Pyakurel A., Glytsou C., Scorrano L. (2020). The cell biology of mitochondrial membrane dynamics. Nat. Rev. Mol. Cell Biol..

[66] Pennanen C., Parra V., López-Crisosto C., Morales P.E., del Campo A., Gutierrez T., Rivera-Mejías P., Kuzmicic J., Chiong M., Zorzano A. (2014). Mitochondrial fission is required for cardiomyocyte hypertrophy mediated by a Ca^2+^-calcineurin signaling pathway. J. Cell Sci..

[67] Ohshima Y., Takata N., Suzuki-Karasaki M., Yoshida Y., Tokuhashi Y., Suzuki-Karasaki Y. (2017). Disrupting mitochondrial Ca^2+^ homeostasis causes tumor-selective TRAIL sensitization through mitochondrial network abnormalities. Int. J. Oncol..

[68] Han X.J., Lu Y.F., Li S.A., Kaitsuka T., Sato Y., Tomizawa K., Nairn A.C., Takei K., Matsui H., Matsushita M. (2008). CaM kinase Iα-induced phosphorylation of Drp1 regulates mitochondrial morphology. J. Cell Biol..

[69] Gomez-Suaga P., Paillusson S., Stoica R., Noble W., Hanger D.P., Miller C.C.J. (2017). The ER-Mitochondria Tethering Complex VAPB-PTPIP51 Regulates Autophagy. Curr. Biol..

[70] Vance J.E. (2014). MAM (mitochondria-associated membranes) in mammalian cells: Lipids and beyond. Biochim. Biophys. Acta Mol. Cell Biol. Lipids.

[71] Gizak A., Pirog M., Rakus D. (2012). Muscle FBPase binds to cardiomyocyte mitochondria under glycogen synthase kinase-3 inhibition or elevation of cellular Ca^2+^ level. FEBS Lett..

[72] Wen Y.-A., Xiong X., Scott T., Li A.T., Wang C., Weiss H.L., Tan L., Bradford E., Fan T.W.M., Chandel N.S. (2019). The mitochondrial retrograde signaling regulates Wnt signaling to promote tumorigenesis in colon cancer. Cell Death Differ..

[73] Delgado-Deida Y., Alula K.M., Theiss A.L. (2020). The influence of mitochondrial-directed regulation of Wnt signaling on tumorigenesis. Gastroenterol. Rep..

[74] Tsai F.C., Seki A., Yang H.W., Hayer A., Carrasco S., Malmersjö S., Meyer T. (2014). A polarized Ca^2+^, diacylglycerol and STIM1 signalling system regulates directed cell migration. Nat. Cell Biol..

[75] Romero-Gar S., Prado-Garcia H. (2019). Mitochondrial calcium: Transport and modulation of cellular processes in homeostasis and cancer. Int. J. Oncol..

[76] Tosatto A., Sommaggio R., Kummerow C., Bentham R.B., Blacker T.S., Berecz T., Duchen M.R., Rosato A., Bogeski I., Szabadkai G. (2016). The mitochondrial calcium uniporter regulates breast cancer progression via HIF-1α. EMBO Mol. Med..

[77] Yu C., Wang Y., Peng J., Shen Q., Chen M., Tang W., Li X., Cai C., Wang B., Cai S. (2017). Mitochondrial calcium uniporter as a target of microRNA-340 and promoter of metastasis via enhancing the Warburg effect. Oncotarget.

[78] Gogvadze V., Zhivotovsky B., Orrenius S. (2010). The Warburg effect and mitochondrial stability in cancer cells. Mol. Aspects Med..

[79] Medes G., Thomas A., Weinhouse S. (1953). Metabolism of Neoplastic Tissue. IV. A Study of Lipid Synthesis in Neoplastic Tissue Slices in Vitro. Cancer Res..

[80] Rusiñol A.E., Cui Z., Chen M.H., Vance J.E. (1994). A unique mitochondria-associated membrane fraction from rat liver has a high capacity for lipid synthesis and contains pre-Golgi secretory proteins including nascent lipoproteins. J. Biol. Chem..

[81] Kainu V., Hermansson M., Hänninen S., Hokynar K., Somerharju P. (2013). Import of phosphatidylserine to and export of phosphatidylethanolamine molecular species from mitochondria. Biochim. Biophys. Acta Mol. Cell Biol. Lipids.

[82] Hernández-Alvarez M.I., Sebastián D., Vives S., Ivanova S., Bartoccioni P., Kakimoto P., Plana N., Veiga S.R., Hernández V., Vasconcelos N. (2019). Deficient Endoplasmic Reticulum-Mitochondrial Phosphatidylserine Transfer Causes Liver Disease. Cell.

[83] Guo D. (2017). Lipid droplets, potential biomarker and metabolic target in glioblastoma. Intern. Med. Rev..

[84] Mulas M.F., Abete C., Pulisci D., Pani A., Massidda B., Dessì S., Mandas A. (2011). Cholesterol esters as growth regulators of lymphocytic leukaemia cells. Cell Prolif..

[85] Antalis C.J., Arnold T., Rasool T., Lee B., Buhman K.K., Siddiqui R.A. (2010). High ACAT1 expression in estrogen receptor negative basal-like breast cancer cells is associated with LDL-induced proliferation. Breast Cancer Res. Treat..

[86] Gaida M.M., Mayer C., Dapunt U., Stegmaier S., Schirmacher P., Wabnitz G.H., Maria Hänsch G. (2016). Expression of the bitter receptor T2R38 in pancreatic cancer: Localization in lipid droplets and activation by a bacteria-derived quorum-sensing molecule. Oncotarget.

[87] Accioly M.T., Pacheco P., Maya-Monteiro C.M., Carrossini N., Robbs B.K., Oliveira S.S., Kaufmann C., Morgado-Diaz J.A., Bozza P.T., Viola J.P.B. (2008). Lipid bodies are reservoirs of cyclooxygenase-2 and sites of prostaglandin-E2 synthesis in colon cancer cells. Cancer Res..

[88] Naon D., Scorrano L. (2014). At the right distance: ER-mitochondria juxtaposition in cell life and death. Biochim. Biophys. Acta Mol. Cell Res..

[89] Stopsack K.H., Gerke T.A., Andrén O., Andersson S.O., Giovannucci E.L., Mucci L.A., Rider J.R. (2017). Cholesterol uptake and regulation in high-grade and lethal prostate cancers. Carcinogenesis.

[90] Li J., Gu D., Lee S.S.Y., Song B., Bandyopadhyay S., Chen S., Konieczny S.F., Ratliff T.L., Liu X., Xie J. (2016). Abrogating cholesterol esterification suppresses growth and metastasis of pancreatic cancer. Oncogene.

[91] Ohmoto T., Nishitsuji K., Yoshitani N., Mizuguchi M., Yanagisawa Y., Saito H., Sakashita N. (2015). K604, a specific acyl-CoA:cholesterol acyltransferase 1 inhibitor, suppresses proliferation of U251-MG glioblastoma cells. Mol. Med. Rep..

[92] Kim M.P., Gallick G.E. (2008). Gemcitabine resistance in pancreatic cancer: Picking the key players. Clin. Cancer Res..

[93] Liscovitch M., Lavie Y. (2000). Multidrug resistance: A role for cholesterol efflux pathways?. Trends Biochem. Sci..

[94] Thoudam T., Ha C.M., Leem J., Chanda D., Park J.S., Kim H.J., Jeon J.H., Choi Y.K., Liangpunsakul S., Huh Y.H. (2019). PDK4 augments ER–mitochondria contact to dampen skeletal muscle insulin signaling during obesity. Diabetes.

[95] Sood A., Jeyaraju D.V., Prudent J., Caron A., Lemieux P., McBride H.M., Laplante M., Tóth K., Pellegrini L. (2014). A Mitofusin-2-dependent inactivating cleavage of Opa1 links changes in mitochondria cristae and ER contacts in the postprandial liver. Proc. Natl. Acad. Sci. USA.

[96] Theurey P., Tubbs E., Vial G., Jacquemetton J., Bendridi N., Chauvin M.A., Alam M.R., Le Romancer M., Vidal H., Rieusset J. (2016). Mitochondria-associated endoplasmic reticulum membranes allow adaptation of mitochondrial metabolism to glucose availability in the liver. J. Mol. Cell Biol..

[97] Carraro R.S., Souza G.F., Solon C., Razolli D.S., Chausse B., Barbizan R., Victorio S.C., Velloso L.A. (2018). Hypothalamic mitochondrial abnormalities occur downstream of inflammation in diet-induced obesity. Mol. Cell. Endocrinol..

[98] Theurey P., Rieusset J. (2017). Mitochondria-Associated Membranes Response to Nutrient Availability and Role in Metabolic Diseases. Trends Endocrinol. Metab..

[99] Horndasch M., Lienkamp S., Springer E., Schmitt A., Pavenstädt H., Walz G., Gloy J. (2006). The C/EBP homologous protein CHOP (GADD153) is an inhibitor of Wnt/TCF signals. Oncogene.

[100] Verras M., Papandreou I., Lim A.L., Denko N.C. (2008). Tumor Hypoxia Blocks Wnt Processing and Secretion through the Induction of Endoplasmic Reticulum Stress. Mol. Cell. Biol..

[101] Morris S.A.L., Huang S. (2016). Crosstalk of the Wnt/β-catenin pathway with other pathways in cancer cells. Genes Dis..

[102] Morris J.P., Wang S.C., Hebrok M. (2010). KRAS, Hedgehog, Wnt and the twisted developmental biology of pancreatic ductal adenocarcinoma. Nat. Rev. Cancer.

[103] Nusse R., Varmus H.E. (1982). Many tumors induced by the mouse mammary tumor virus contain a provirus integrated in the same region of the host genome. Cell.

[104] Korinek V., Barker N., Morin P.J., Van Wichen D., De Weger R., Kinzler K.W., Vogelstein B., Clevers H. (1997). Constitutive transcriptional activation by a β-catenin-Tcf complex in APC(-/-) colon carcinoma. Science.

[105] Duchartre Y., Kim Y.M., Kahn M. (2016). The Wnt signaling pathway in cancer. Crit. Rev. Oncol. Hematol..

[106] Zhan T., Rindtorff N., Boutros M. (2017). Wnt signaling in cancer. Oncogene.

[107] Bernkopf D.B., Jalal K., Brückner M., Knaup K.X., Gentzel M., Schambony A., Behrens J. (2018). Pgam5 released from damaged mitochondria induces mitochondrial biogenesis via Wnt signaling. J. Cell Biol..

[108] Bernkopf D.B., Behrens J. (2018). Feedback regulation of mitochondrial homeostasis via Wnt/β-catenin signaling. Mol. Cell. Oncol..

[109] Woo D.K., Green P.D., Santos J.H., D’Souza A.D., Walther Z., Martin W.D., Christian B.E., Chandel N.S., Shadel G.S. (2012). Mitochondrial genome instability and ROS enhance intestinal tumorigenesis in APC Min/+ mice. Am. J. Pathol..

[110] Xia Z., Wu S., Wei X., Liao Y., Yi P., Liu Y., Liu J., Liu J. (2019). Hypoxic ER stress suppresses β-catenin expression and promotes cooperation between the transcription factors XBP1 and HIF1α for cell survival. J. Biol. Chem..

[111] Zhang L.S., Lum L. (2018). Chemical Modulation of WNT Signaling in Cancer. Progress in Molecular Biology and Translational Science.

[112] Shikata Y., Kiga M., Futamura Y., Aono H., Inoue H., Kawada M., Osada H., Imoto M. (2017). Mitochondrial uncoupler exerts a synthetic lethal effect against β-catenin mutant tumor cells. Cancer Sci..

[113] Ishii I., Harada Y., Kasahara T. (2012). Reprofiling a classical anthelmintic, pyrvinium pamoate, as an anti-cancer drug targeting mitochondrial respiration. Front. Oncol..

[114] Thorne C.A., Hanson A.J., Schneider J., Tahinci E., Orton D., Cselenyi C.S., Jernigan K.K., Meyers K.C., Hang B.I., Waterson A.G. (2010). Small-molecule inhibition of Wnt signaling through activation of casein kinase 1α. Nat. Chem. Biol..

[115] Li J., He X., Zou Y., Chen D., Yang L., Rao J., Chen H., Chan M.C.W., Li L., Guo Z. (2017). Mitochondria-targeted platinum(II) complexes: Dual inhibitory activities on tumor cell proliferation and migration/invasion: Via intracellular trafficking of β-catenin. Metallomics.

[116] Nangia-Makker P., Yu Y., Vasudevan A., Farhana L., Rajendra S.G., Levi E., Majumdar A.P.N. (2014). Metformin: A potential therapeutic agent for recurrent colon cancer. PLoS ONE.

[117] Fennelly C., Amaravadi R.K. (2017). Lysosomal Biology in Cancer. Methods Mol. Biol..

[118] Guicciardi M.E., Leist M., Gores G.J. (2004). Lysosomes in cell death. Oncogene.

[119] Nylandsted J., Gyrd-Hansen M., Danielewicz A., Fehrenbacher N., Lademann U., Høyer-Hansen M., Weber E., Multhoff G., Rohde M., Jäättelä M. (2004). Heat shock protein 70 promotes cell survival by inhibiting lysosomal membrane permeabilization. J. Exp. Med..

[120] Kirkegaard T., Jäättelä M. (2009). Lysosomal involvement in cell death and cancer. Biochim. Biophys. Acta Mol. Cell Res..

[121] Madge L.A., Li J.H., Choi J., Pober J.S. (2003). Inhibition of phosphatidylinositol 3-kinase sensitizes vascular endothelial cells to cytokine-initiated cathepsin-dependent apoptosis. J. Biol. Chem..

[122] Saxton R.A., Sabatini D.M. (2017). mTOR Signaling in Growth, Metabolism, and Disease. Cell.

[123] Hua H., Kong Q., Zhang H., Wang J., Luo T., Jiang Y. (2019). Targeting mTOR for cancer therapy. J. Hematol. Oncol..

[124] Sterea A.M., Almasi S., El Hiani Y. (2018). The hidden potential of lysosomal ion channels: A new era of oncogenes. Cell Calcium.

[125] Xu H., Ren D. (2015). Lysosomal Physiology. Annu. Rev. Physiol..

[126] Tong Y., Song F. (2015). Intracellular calcium signaling regulates autophagy via calcineurinmediated TFEB dephosphorylation. Autophagy.

[127] Li R.J., Xu J., Fu C., Zhang J., Zheng Y.G., Jia H., Liu J.O. (2016). Regulation of mTORC1 by lysosomal calcium and calmodulin. Elife.

[128] Zhu M.X. (2017). A well-known potassium channel plays a critical role in lysosomes. J. Cell Biol..

[129] Vyas S., Zaganjor E., Haigis M.C. (2016). Mitochondria and Cancer. Cell.

[130] Viale A., Pettazzoni P., Lyssiotis C.A., Ying H., Sanchez N., Marchesini M., Carugo A., Green T., Seth S., Giuliani V. (2014). Oncogene ablation-resistant pancreatic cancer cells depend on mitochondrial function. Nature.

[131] Palikaras K., Tavernarakis N. (2014). Mitochondrial homeostasis: The interplay between mitophagy and mitochondrial biogenesis. Exp. Gerontol..

[132] Wong Y.C., Kim S., Peng W., Krainc D. (2019). Regulation and Function of Mitochondria-Lysosome Membrane Contact Sites in Cellular Homeostasis. Trends Cell Biol..

[133] Eisenberg-Bord M., Shai N., Schuldiner M., Bohnert M. (2016). A Tether Is a Tether Is a Tether: Tethering at Membrane Contact Sites. Dev. Cell.

[134] Wong Y.C., Ysselstein D., Krainc D. (2018). Mitochondria-lysosome contacts regulate mitochondrial fission via RAB7 GTP hydrolysis. Nature.

[135] Onoue K., Jofuku A., Ban-Ishihara R., Ishihara T., Maeda M., Koshiba T., Itoh T., Fukuda M., Otera H., Oka T. (2013). Fis1 acts as a mitochondrial recruitment factor for TBC1D15 that is involved in regulation of mitochondrial morphology. J. Cell Sci..

[136] Pankiv S., Alemu E.A., Brech A., Bruun J.A., Lamark T., Øvervatn A., Bjørkøy G., Johansen T. (2010). FYCO1 is a Rab7 effector that binds to LC3 and PI3P to mediate microtubule plus end—Directed vesicle transport. J. Cell Biol..

[137] Jordens I., Fernandez-Borja M., Marsman M., Dusseljee S., Janssen L., Calafat J., Janssen H., Wubbolts R., Neefjes J. (2001). The Rab7 effector protein RILP controls lysosomal transport by inducing the recruitment of dynein-dynactin motors. Curr. Biol..

[138] Scorrano L. (2013). Keeping mitochondria in shape: A matter of life and death. Eur. J. Clin. Investig..

[139] Brahimi-Horn M.C., Lacas-Gervais S., Adaixo R., Ilc K., Rouleau M., Notte A., Dieu M., Michiels C., Voeltzel T., Maguer-Satta V. (2015). Local Mitochondrial-Endolysosomal Microfusion Cleaves Voltage-Dependent Anion Channel 1 To Promote Survival in Hypoxia. Mol. Cell. Biol..

[140] Peng W., Wong Y.C., Krainc D. (2020). Mitochondria-lysosome contacts regulate mitochondrial Ca(2+) dynamics via lysosomal TRPML1. Proc. Natl. Acad. Sci. USA.

[141] Schrader M., Costello J., Godinho L.F., Islinger M. (2015). Peroxisome-mitochondria interplay and disease. J. Inherit. Metab. Dis..

[142] Cohen Y., Klug Y.A., Dimitrov L., Erez Z., Chuartzman S.G., Elinger D., Yofe I., Soliman K., Gärtner J., Thoms S. (2014). Peroxisomes are juxtaposed to strategic sites on mitochondria. Mol. Biosyst..

[143] Hosoi K.I., Miyata N., Mukai S., Furuki S., Okumoto K., Cheng E.H., Fujiki Y. (2017). The VDAC2-BAK axis regulates peroxisomal membrane permeability. J. Cell Biol..

[144] Thoms S., Grønborg S., Gärtner J. (2009). Organelle interplay in peroxisomal disorders. Trends Mol. Med..

[145] Fransen M., Lismont C., Walton P. (2017). The peroxisome-mitochondria connection: How and why?. Int. J. Mol. Sci..

[146] Tang Y., He Y., Zhang P., Wang J., Fan C., Yang L., Xiong F., Zhang S., Gong Z., Nie S. (2018). LncRNAs regulate the cytoskeleton and related Rho/ROCK signaling in cancer metastasis. Mol. Cancer.

[147] Walker C.L., Pomatto L.C.D., Tripathi D.N., Davies K.J.A. (2018). Redox regulation of homeostasis and proteostasis in peroxisomes. Physiol. Rev..

[148] Wei F., Tang L., He Y., Wu Y., Shi L., Xiong F., Gong Z., Guo C., Li X., Liao Q. (2018). BPIFB1 (LPLUNC1) inhibits radioresistance in nasopharyngeal carcinoma by inhibiting VTN expression. Cell Death Dis..

[149] Schieber M., Chandel N.S. (2014). ROS function in redox signaling and oxidative stress. Curr. Biol..

[150] Diebold L., Chandel N.S. (2016). Mitochondrial ROS regulation of proliferating cells. Free Radic. Biol. Med..

[151] He Y., Jing Y., Wei F., Tang Y., Yang L., Luo J., Yang P., Ni Q., Pang J., Liao Q. (2018). Long non-coding RNA PVT1 predicts poor prognosis and induces radioresistance by regulating DNA repair and cell apoptosis in nasopharyngeal carcinoma. Cell Death Dis..

[152] Raza M.H., Siraj S., Arshad A., Waheed U., Aldakheel F., Alduraywish S., Arshad M. (2017). ROS-modulated therapeutic approaches in cancer treatment. J. Cancer Res. Clin. Oncol..

[153] Fulda S., Galluzzi L., Kroemer G. (2010). Targeting mitochondria for cancer therapy. Nat. Rev. Drug Discov..

[154] Leanza L., Romio M., Becker K.A., Azzolini M., Trentin L., Managò A., Venturini E., Zaccagnino A., Mattarei A., Carraretto L. (2017). Direct Pharmacological Targeting of a Mitochondrial Ion Channel Selectively Kills Tumor Cells In Vivo. Cancer Cell.

